# Cost-effectiveness analysis of Coblation versus mechanical shaver debridement in patients following knee chondroplasty

**DOI:** 10.1186/s12962-020-00240-w

**Published:** 2020-10-16

**Authors:** Ayoade Adeyemi, Leo Nherera, Paul Trueman, Anil Ranawat

**Affiliations:** 1 Smith & Nephew, Inc, Andover, MA USA; 2 Smith & Nephew, Inc, Hull, UK; 3 Department of Sports, Hospital for Special Surgery, New York, NY USA; 4 Smith & Nephew Inc, 5600 Clearfork Main Street, Fort Worth, TX 76109 USA

**Keywords:** Coblation, Chondroplasty, Mechanical shaver debridement, Cost-effectiveness, Revision rates, Cartilage lesions

## Abstract

**Background:**

To compare costs and outcomes following knee chondroplasty with Coblation versus mechanical shaver debridement (MSD) in patients with grade III articular cartilage lesions of the knee.

**Methods:**

A decision-analytic model was developed to compare costs and outcomes of the two methods from a US payer perspective. We used published clinical data from a single-center randomized clinical trial (RCT) designed to compare outcomes between Coblation and MSD in patients with grade III articular cartilage lesions of the medial femoral condyle. Following primary knee chondroplasty, patients experienced either treatment success (no additional surgery required) or required a revision over the 4 year follow-up period. Costs associated with the initial chondroplasty, physical therapy sessions through the 6 week postoperative period, and revision rates at 4 years post-surgery were estimated using 2018 US Medicare Physician Fee Schedule. Sensitivity analyses including a 10 year time horizon and threshold analyses were performed to test the robustness of the model.

**Results:**

The estimated total cost per patient was $4614 and $7886 for Coblation and MSD, respectively, resulting in cost-savings of $3272 in favor of Coblation, making it a dominant strategy because of lower costs and improved clinical outcomes. Threshold analysis showed that Coblation remained dominant even when revision rates were assumed to increase from the base case rate of 14–66%. Sensitivity analyses showed that cost-saving results were insensitive to variations in revision rates, number of physical therapy sessions and the time horizon used.

**Conclusion:**

Coblation chondroplasty is a cost-saving procedure compared with MSD in the treatment of patients with grade III articular cartilage lesions of the knee.

## Introduction

Chondral defects of the knee joint are common [1, 2] and may result in functional impairment and adversely affect quality of life, generating considerable costs to society [3]. Of knee articular cartilage injuries evaluated during arthroscopic procedures, approximately 63% are identified as chondral lesions, with more than 40% of these classified as grade III based on the International Cartilage Research Society (ICRS) grading system [4]. Patients with grade III chondral lesions have deep crevices that are greater than 50% of the cartilage but not full thickness and are associated with gross fibrillations, fissuring and rough surfaces [5]. These cartilage defects may eventually progress to osteoarthritis and joint degeneration [6]. In 2008, it was estimated that 27 million adults in the United States presented with osteoarthritis [7]. In 2009, about 905,000 knee and hip replacements were performed in the United States at an estimated treatment cost of $42.3 billion [8]. Interventions that repair or treat cartilage defects may help reduce the economic burden of osteoarthritis. These interventions include surgical procedures which until as recent as 2007, had not received the deserved attention due to perceived lack of cost-effectiveness evidence [9].

For cartilage lesions not amenable to repair, chondroplasty (cartilage repair) is a viable and common treatment option. Traditionally, mechanical shavers have been used for chondral debridement, often resulting in the removal of healthy underlying tissue and, consequently, undesirable adverse effects [10, 11]. Numerous studies have reported the potential benefits of radiofrequency-based ablation therapies for knee chondroplasty [10, 14]. These studies have shown that radiofrequency-based therapies prevent damage to the cartilage and provide a smoothing effect without further fibrillation. Traditionally, surgeons have avoided radio-frequency based therapies because of fears of chondrocyte death from high temperature [11, 12, 16–20].

Coblation (COBLATION™ Plasma Technology; Smith & Nephew, Fort Worth, TX, USA) is a controlled ablation technology that uses a stable, low-temperature plasma field to precisely remove devitalized tissue [21, 24]. When used to treat diseased cartilage, Coblation effectively removes defective cartilage with great accuracy and precision affording minimal damage to the perilesional cartilage [5, 25]. Results of a randomized clinical trial (RCT) show a significantly lower revision rate with Coblation compared with mechanical shaver debridement (MSD) (14 vs 48%, respectively, *P* = 0.006) [25]. Furthermore the 10 year follow-up of the same study confirmed that revision rates with Coblation remained low at 24% compared to 62% for patients treated with MSD [25]. In addition, patients treated with Coblation report greater improvements in their symptoms, knee pain, and physical activity as demonstrated by significantly higher Knee Injury and Osteoarthritis Outcome Scores (KOOS) at the end of the study [25]. However, it remains unclear to what extent these favorable clinical outcomes impact healthcare costs. Thus, the purpose of this study was to compare the costs and outcomes of knee cartilage repair with Coblation to those with MSD in the short term using the 4 year RCT data in the base case analysis.

## Methods

We evaluated the costs and outcomes associated with Coblation compared with MSD following a knee chondroplasty procedure. The clinical outcomes data for this economic analysis were extracted from a single-center RCT over 1, 4 and 10 year follow-up periods involving 60 patients with a medial meniscus tear and an idiopathic grade III cartilage defect of the medial femoral condyle, confirmed by radiography and magnetic resonance imaging [5, 25, 26]. The Spahn study was chosen because it is currently the only RCT that directly compares the effectiveness of Coblation and MSD over time after the initial knee cartilage repair procedure. In sensitivity analyses, we included the 10 year follow-up data to assess the long-term cost-effectiveness of the interventions.

In the RCT, patients and the investigator who assessed the radiographs were blinded to treatment assignments through the follow-up periods. Patients were excluded if they had a history of major knee injuries, previous surgeries, knee osteoarthritis, and at least a grade II cartilage defect of the tibial joint surface on the lateral compartment of the femoro-patellar joint. Cost data were extracted from the 2018 US Medicare Physician Fee Schedule, [27] these included payments for inpatient, outpatient, and provider-related services; trial-related costs were excluded from cost estimation in this analysis in order to appropriately capture the costs associated with knee cartilage lesion repair in actual practice. The time horizon for the base case analysis was 4 years for both costs and outcomes and extended to 10 years in sensitivity analysis. This study was based on an economic model that was developed using published clinical aggregate data; hence, Institutional Review Board approval was not required.

### Economic model

**Fig. 1:**
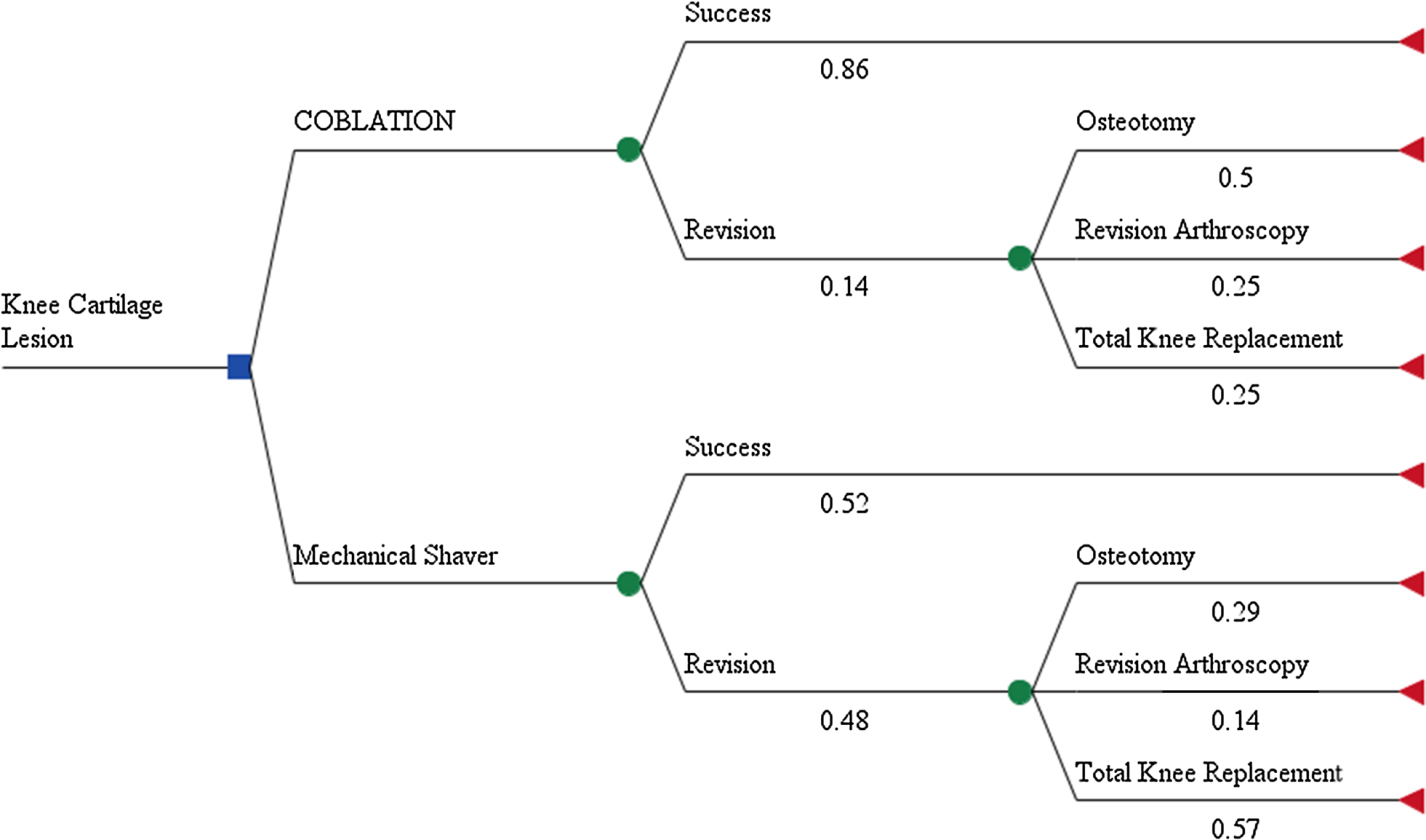
Decision model comparing outcomes between Coblation and mechanical shaver debridement (MSD) Chondroplasty. The decision tree starts with the option of treatment of knee cartilage lesion with Coblation or MSD represented by a square. The chance nodes (circles) represent the probability of success or a need for revision of the treatment and subsequently down the tree the probability of treatment revisions

A simple decision tree was developed to compare the costs and clinical outcomes of Coblation versus MSD following knee chondroplasty from the US payer perspective (Fig. 1). The decision tree illustrates a cohort of patients undergoing knee chondroplasty with Coblation or MSD. Following the primary knee chondroplasty procedure, patients experience a treatment success or are assumed to require secondary surgery (hereafter, referred to as revision). Patients requiring a revision are identified as those who had a revision procedure in the trial (Fig. 1). These revision procedures included revision arthroscopy, osteotomy, or total knee replacement. All analyses were conducted in Microsoft Excel (2013 Version 15.0).

### Patient characteristics

Based on the single-center RCT, a total of 30 patients were initially included in each of the Coblation and MSD groups; by the 4-year postoperative follow-up period, 1 patient was lost to follow-up per group. Baseline characteristics between the Coblation and MSD groups were comparable (mean age: 42.9 vs 43.7 years, percentage of males: 43% vs 50%; injury onset: 8.4 vs 7.4 months; body mass index: 26.6 vs 27.8 kg/m [2]; normalized KOOS: 10.8 vs 10.3 points) [5]. Other baseline clinical characteristics are described in greater detail in the published results of the RCT. [5, 25].

### Clinical outcome

The main outcome measure used in this study was defined as the revision rate. The overall probability of requiring a revision and the probabilities of having a revision arthroscopy, an osteotomy, or a total knee replacement at 4 years following the initial knee chondroplasty procedure were taken from the RCT [25] after accounting for 2 patients lost to follow-up (one from each treatment group [1 patient from the Mechanical shaver group had died and 1 patient from the Coblation group was lost to follow-up] and applied to the model. The RCT reported a lower revision rate with Coblation, 14% compared with 48% with MSD. Of the 18 patients who had revisions for persistent knee problems, there were 8 total knee replacements, 4 osteotomies, and 2 arthroscopies in the MSD group; and 1 total knee replacement, 2 osteotomies, and 1 arthroscopy in the Coblation group [25]. In addition, patients treated with Coblation reported a significantly higher KOOS at the end of the follow-up period (71.8 points vs. 53.2 points, respectively; *P* < 0.001), see Table 1. At the 10 year follow-up, an additional 7 patients required revision surgery, there were 3 unicondylar/total knee replacements and 1 osteotomy procedure required in the MSD group while 3 revision procedures were required in the Coblation group [26]. Although results of the RCT did not state the specific revision procedures required in the Coblation group, a worst case scenario was assumed and total knee replacement procedures were assumed for all 3 revision procedures. Furthermore, with comparable baseline KOOS scores, the Coblation group had a significantly higher KOOS score compared to the MSD group (50.8 points vs. 33.1 points, respectively; *p* < 0.001).

**Table 1:** Revisions and clinical outcomes at 4 year follow-up

Outcomes^a^	COBLATION	MSD
Overall revisions N (%)	4 (0.14)	14 (0.48)
Specific revisions N (%)		
Osteotomy	2 (0.07)	4 (0.14)
Arthroscopy	1 (0.03)	2 (0.07)
Total Knee Arthroplasty	1 (0.03)	8 (0.28)
Pre-operative Outcome KOOS	15.50 (12.7)	11.30 (8.8)
Post-operative Outcome KOOS^b^	71.80 (9.1)	53.20 (17.5)

*KOOS* Knee Injury and Osteoarthritis Outcome Score; *MSD* mechanical shaver debridement; *TKA* total knee replacement

^a^Outcomes are from the randomized controlled trial [25]

^b^Statistically significant difference in post-operative KOOS score between Coblation and MSD (*p* value < 0.001)

### Costs

This study utilized the 2018 US Medicare Physician Fee Schedule to estimate the costs associated with the initial chondroplasty procedure, physical therapy sessions through the 6 week postoperative period, and revisions at 4 years post-surgery [27]. Costs included were inpatient/outpatient facility and provider payments for the services of interest. Total primary procedure costs were the sum of the costs for the initial procedure and physical therapy sessions used through the 6 week period post-surgery. Revision costs associated with reimbursement (payments) for arthroscopic revision, osteotomy, and total knee replacement based on relevant diagnostic-related groups and current procedural terminology codes were used to estimate total cost per patient over the 4 year follow-up period, see Table 2. The procedure cost for chondroplasty was $3207. In accordance with recommendations of the Panel on Cost-Effectiveness in Health and Medicine, an annual discount rate of 3% was applied to the revision costs over 4 years [28]. The estimated reimbursements for arthroscopic revision, osteotomy and knee replacement 4 years following the initial arthroscopic procedure were $2935, $5855 and $10,553 respectively. The cost of a physiotherapy session was $82 and the number of physiotherapy sessions was taken from the RCT by Spahn [25]. All other resources used such as durable medical equipment, pain medications and prophylactic antibiotics were assumed to be comparable between the two treatment interventions and therefore were not explicitly costed.

**Table 2:** Procedure description and related current procedural terminology and diagnosis-related group codes

Description of procedure	CPT code	APC/DRG codes used
Arthroscopy, knee, surgical; with meniscectomy (medial *or* lateral, including any meniscal shaving) including debridement/shaving of articular cartilage (chondroplasty), same or separate compartment(s), when performed	29,881	5113
Osteotomy	27,705	494
Total knee replacement	27,447	470

*APC* ambulatory payment classification; *CPT* current procedural terminology; *DRG* diagnosis-related group without complications

### Cost-effectiveness and sensitivity analyses

A cost-effectiveness ratio, defined as the ratio of the difference in total costs and the difference in number of revisions between Coblation and MSD, was estimated using the rollback method. To assess uncertainties and account for possible sources of bias in the model, multiple one-way sensitivity analyses were conducted. Based on the reference case, reported distributions (standard deviations) were applied to the base case estimates of physical therapy sessions. Because of the lack of published data on revisions in the literature, we assumed that the revision rates could vary by 50% above and below the reported rates from the RCT, this variation was also accounted for in the corresponding success rate. A threshold analysis was performed to determine the revision rate at which the use of Coblation became cost-neutral compared with the use of MSD. This involved increasing the revision rate of Coblation from the initially reported rate until the cost of the Coblation procedure was equivalent to that of MSD. We also assessed the long-term costs and outcomes of the interventions using the 10 year follow-up data, [26] thus extending the time horizon and discounting to 10 years. All relevant input parameters used in the cost-effectiveness model, including the range of values used in the sensitivity analyses are shown in Table 3.

**Table 3:** Inputs and ranges for all parameters in the cost-effectiveness model at 4 year follow-up

	Base case^a^	Range^b^	
		Low	High
Cumulative 4 year probabilities^a^			
Probability of treatment success			
Coblation	0.86	0.79	0.93
MSD	0.52	0.28	0.76
Probability of revision (1-treatment success)			
Coblation	0.14	0.07	0.21
MSD	0.48	0.24	0.72
Probability of revision procedures with Coblation			
Osteotomy	0.50	0.25	0.75
Revision arthroscopy	0.25	0.13	0.38
Total knee replacement	0.25	0.13	0.38
Probability of revision procedure with MSD			
Osteotomy	0.29	0.15	0.44
Revision arthroscopy	0.14	0.07	0.21
Total knee replacement	0.57	0.29	0.86
Number of physical therapy sessions used^b^			
Coblation	6.4	4.8	8.0
MSD	9.8	9.2	10.4
Costs, 2018 (US$)^c^			
Primary knee chondroplasty	3207		
Physical therapy session	82	–	–
4 year revision procedures			
Osteotomy	5855	–	–
Revision arthroscopy	2935	–	–
Total knee replacement	10,553	–	–

*MSD* mechanical shaver debridement

^a^Based on published results of the randomized clinical trial [25]

^b^Base case revision probabilities were varied by 50%, except for number of physical therapy sessions which were based on randomized clinical trial [5]

^c^Based on 2018 US Medicare Physician Fee Schedule [27]

## Results

Over the 4 year post-operative period, the model estimated that the total cost per patient was $4614 for patients treated with Coblation and $7886 for patients treated with MSD resulting in cost-savings of $3272 in favor of Coblation. The main cost driver in this model was the lower revision rate in patients treated with Coblation compared to MSD, 4 compared to 14 revisions for Coblation and MSD respectively. In economic terms, Coblation is considered a dominant strategy as it results in improved clinical outcomes (significantly lower revision rate) and lower total treatment costs, see Table 4.

**Table 4:** Estimated total costs per patient and outcomes for MSD and Coblation chondroplasty at 4 year follow-up

Intervention	Total Costs (US$)	Revisions	Revision Difference	Cost Difference	ICER
MSD	$7886	14			Dominant
Coblation	$4614	4	− 10	− $3272	

*ICER* incremental cost-effectiveness ratio; *MSD* mechanical shaver debridement

**Fig. 2:**
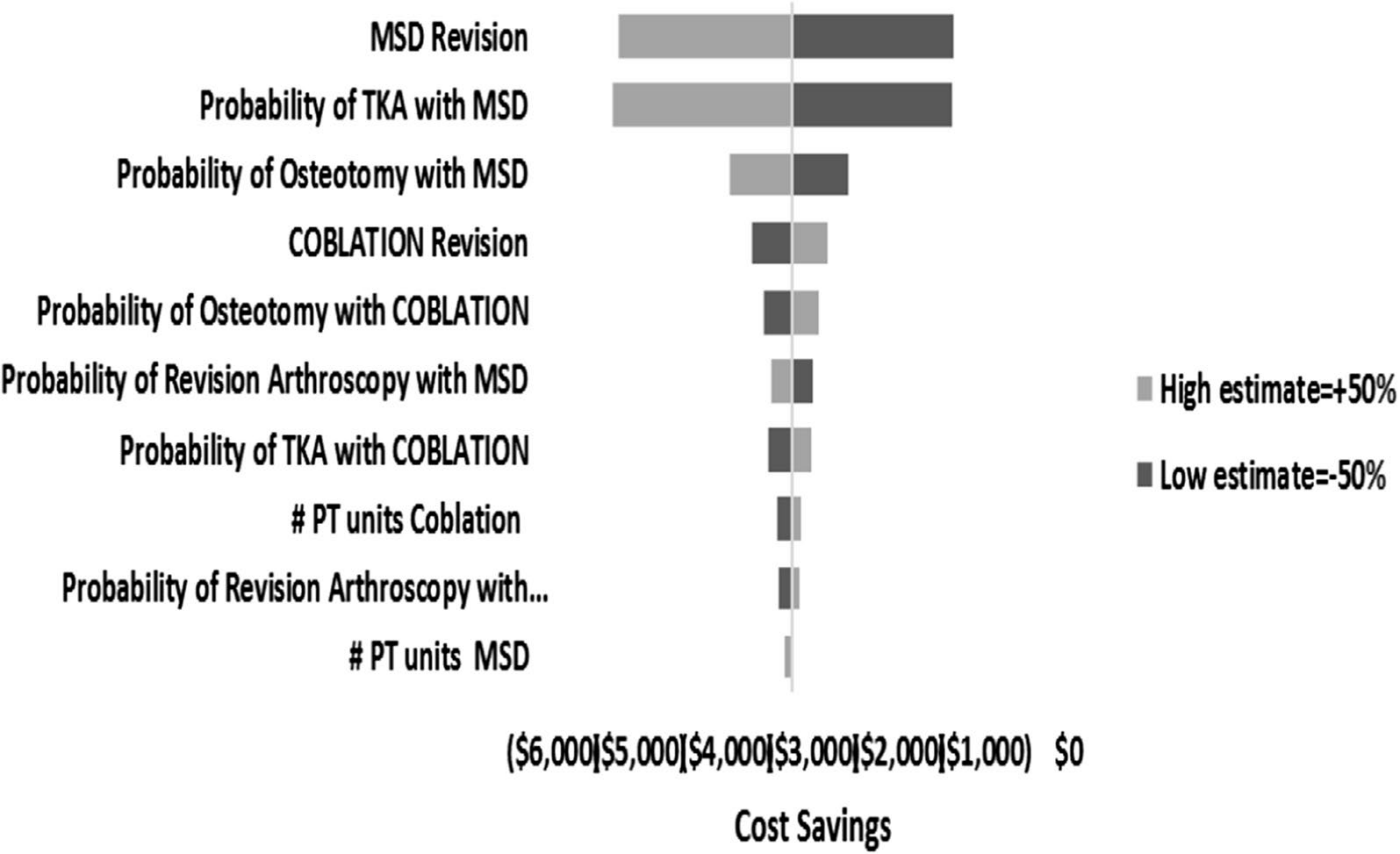
Tornado diagram of the impact of multiple one-way sensitivity analyses on costs associated with Coblation versus MSD chondroplasty. PT unit variations were based on published randomized controlled trial results. *MSD* mechanical shaver debridement, *PT* physical therapy, *TKA* total knee arthroscopy

To account for uncertainties in the model parameters, we conducted a series of one-way sensitivity analyses, see Fig. 2. The long-term 10 year results, see Tables 5 and 6 showed a trend similar to the short term 4 year results and Coblation remained cost saving ($3451 savings per patient). At the 10 year follow-up, the revision rate in the Coblation group was 24% compared to 62% in the MSD group while the estimated total cost for Coblation was $5536 compared to $8986 for MSD.

**Table 5:** Inputs and ranges for parameters in the 10 year cost-effectiveness model

	Base case^a^	Range^b^	
		Low	High
Probability of treatment success			
Coblation	0.76	0.64	0.88
MSD	0.38	0.07	0.69
Probability of revision (1- treatment success)			
Coblation	0.24	0.12	0.36
MSD	0.62	0.31	0.93
Costs, 2018 (US$)^c^			
10 year revision procedures			
Osteotomy	4904	–	–
Revision arthroscopy	2458	–	–
Total knee replacement	8838	–	–

*MSD* mechanical shaver debridemen0074

^a^Based on published results of the randomized clinical trial [25, 26]

^b^10 year revision probabilities were varied by 50%, except for number of physical therapy sessions which were based on randomized clinical trial [5, 25, 26]

^c^Based on 2018 US Medicare Physician Fee Schedule [27]

**Table 6:** Estimated total costs per patient and outcomes for MSD and Coblation chondroplasty at 10-year follow-up

Intervention	Total costs (US$)	Revisions	Revision difference	Cost difference	ICER
MSD	$8986	18			Dominant
Coblation	$5535	7	7	− $3451	

*ICER* incremental cost-effectiveness ratio; *MSD* mechanical shaver debridement

**Fig. 3:**
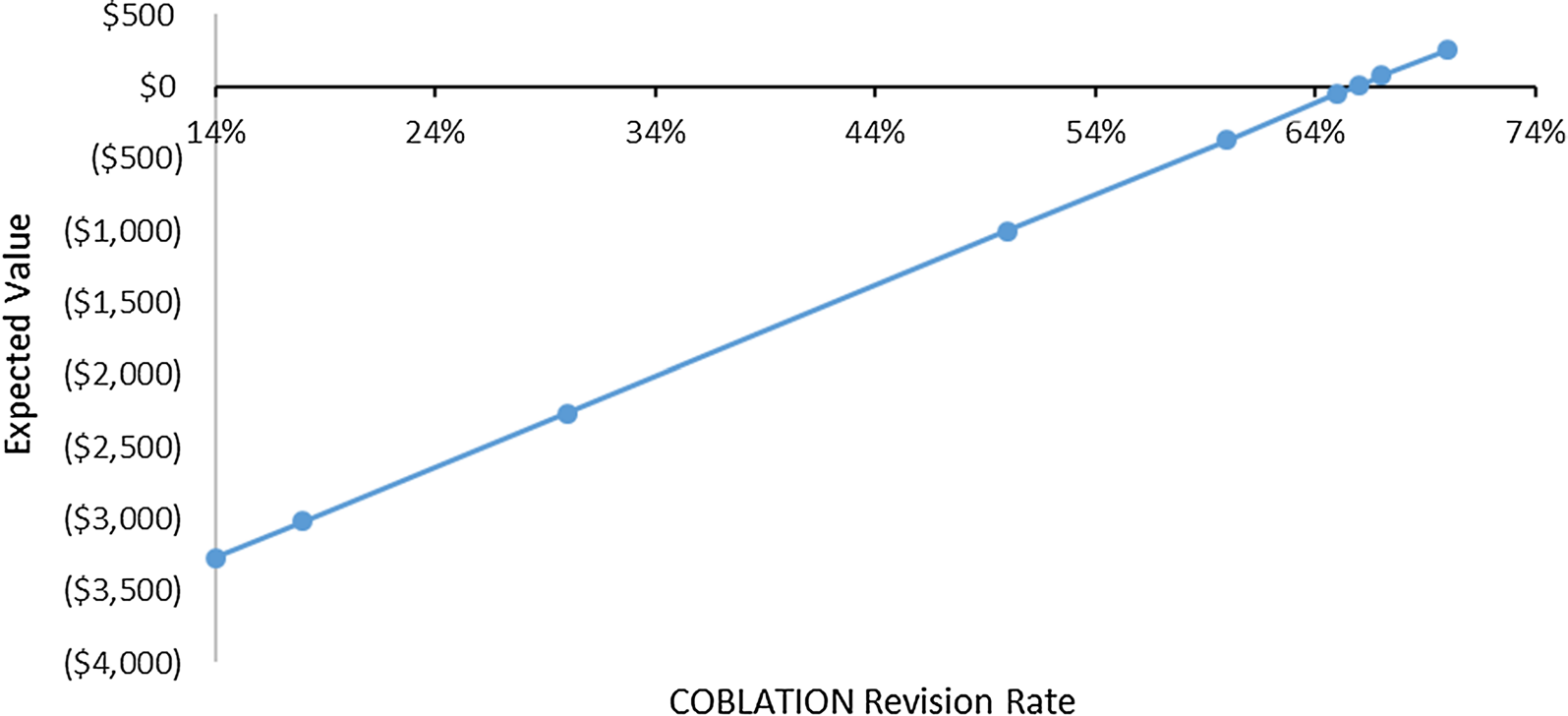
Threshold analysis. This analysis was performed to determine the revision rate at which the use of Coblation becomes cost-neutral compared with the use of mechanical shaver debridement

Revision rates were varied 50% above and below the base case revision rates. Standard deviations around the base case numbers of physical therapy sessions were also applied. Despite these variations, Coblation remained a cost-saving procedure compared with MSD (that is, the model was robust to sensitivity analyses) within the range of model inputs specified. The tornado diagram in Fig. 2 illustrates the impact of various one-way sensitivity analyses on costs. The threshold analysis shows that the use of Coblation remained a cost-neutral procedure compared with MSD even when the initial revision rate of 14% was assumed to increase to 66% (Fig. 3).

## Discussion

Better clinical outcomes (fewer revisions, better functional improvements, less knee pain) in the treatment of grade III articular cartilage lesions were observed with Coblation compared with MSD [25]. The decision-analytic model and analyses described in the present study was designed to compare the costs and outcomes of these two knee chondroplasty procedures. Results of our cost-effectiveness analysis showed that the use of Coblation compared with MSD for knee chondroplasty in patients with a medial meniscus tear and grade III lesions resulted in overall cost-savings to payers of $3272 per patient over a 4 year follow-up period, primarily because of the lower revision rates reported in the RCT by Spahn et al. [5, 25]. Sensitivity analyses performed to test the robustness of the base case results revealed that the observed results remained cost-saving despite variations in key input parameters [26]. Although 10 (16.6%) patients were lost to follow-up at the 10 year follow-up period, decreasing the study power of the RCT to 0.69, an analysis was conducted to examine whether Coblation remained cost-saving at the 10 year follow-up period. Results showed that the use of Coblation ($5535) vs. MSD ($8986) resulted in cost-savings of $3451 over 10 years following the initial chondroplasty procedure due to a lower revision rate, see Tables 5 and 6. The present study utilized both the economic costs of real-world treatment practices and clinical outcome results reported in an RCT and demonstrated long-term cost savings. The use of outcomes data from an RCT that randomly assigned patients to treatment groups such that all measured and unmeasured confounding baseline factors between groups were comparable strengthens the relationship between the interventions used and observed outcomes.

The short-term financial perspective of many healthcare systems may encourage economical behaviors that favor the seemingly least costly intervention. However, our analysis shows the potential for significant cost-savings when decision-makers adopt a longer-term perspective taking into consideration the revision procedures and associated costs that may be avoided in this patient population based on the choice of the initial chondroplasty procedure performed. With the increased emphasis on value-based healthcare, there are indications that healthcare payers are starting to adopt a longer time horizon when considering the cost-effectiveness of new technologies. For example, financial incentives such as restricting payment for avoidable readmissions and bundled payments that cover a 90 day period following surgery both promote consideration of costs beyond the initial acquisition price of an innovative technology [29–31]. Although Coblation represents an incremental cost to healthcare providers undertaking chondroplasty when compared with MSD, our analysis suggests that the incremental costs can be readily offset as a result of improved outcomes, in particular the avoidance of repeat surgery. Healthcare payers should also consider the potential to deliver improved patient outcomes (as illustrated by the KOOS scores reported herein) through the use of Coblation in addition to improved efficiency.

A plausible reason for the improved clinical outcomes observed with Coblation lies in the precise and efficiently controlled ablation process under low-thermal conditions. This controlled ablation process not only eliminates heat-related cell death of healthy chondrocytes that can occur with uncontrolled radiofrequency-based techniques [12, 16, 17, 32] but also prevents the damage and removal of underlying healthy cartilage seen with MSD [10]. The after-effect of damage to healthy cartilage can be a faster onset of the incidence of osteoarthritis, higher revision rates, and the need for procedures such as osteotomy and total knee replacement to address the discomfort and pain associated with osteoarthritis, a chronic and gradually progressive disease. There is evidence to show that loss of knee cartilage volume is associated with significant pain, functional disability, and the onset of knee osteoarthritis that eventually results in total knee replacement within 4 years in a significant proportion of patients [6, 33]. To our knowledge, this is the first cost-effectiveness analysis comparing Coblation with MSD in patients with grade III articular cartilage lesions. The strength of this study lies in the use of clinical outcomes data over a 10 year follow-up period based on the highest level of clinical evidence, an RCT. Furthermore, the conclusions obtained following model adaptations to the German, United Kingdom (UK), and Spanish healthcare systems (data not shown) maintained that Coblation was a cost-saving procedure compared with MSD.

### Limitations

There are some limitations that should be kept in mind when interpreting the results of this study. The costs were based on US Medicare Physician Fee Schedule and are, therefore, more relevant to the US healthcare system. However, similar cost-saving conclusions were realized following model adaptations to the German, UK, and Spanish healthcare systems. Although the inclusion of the hospital and societal perspectives are generally recommended, the pragmatic approach taken in the development of this model is based on available data. The ability to generalize clinical and economic outcomes is an important issue in health economic evaluation as there may be substantial differences in both treatment patterns and the nature of cost savings across countries owing to the differences in healthcare systems. Reimbursement systems, relative prices, and treatment practices, are important issues that vary from country to country and should always be considered.

Furthermore, our model is based on clinical data from a single-center RCT conducted in German patients, the extent to which the patients and the practices adopted in this study reflect real-world practice should be considered. The only complication considered in this model was revision, which is a major cost driver. Using revision as the only complication, favors the intervention that has the greatest impact in reducing this negative outcome in this case Coblation. However, other complications or adverse events were not included in the model as none of these events were observed in the clinical trial data used. Furthermore, we did not discount effectiveness because these effects occurred in the same period between treatment groups and the direction of results would have remained consistent regardless of whether or not effectiveness was discounted. In spite of these limitations, this analysis provides additional insight into the benefits of Coblation relative to MSD in chondroplasty as it supplements the previously reported RCT data on clinical outcomes and patient benefits [5, 25].

## Conclusions

This economic analysis suggests that Coblation chondroplasty is a cost-effective option compared with MSD in the treatment of patients with a medial meniscus tear and idiopathic ICRS grade III defect. Improved outcomes and lower total treatment costs over up to a 10 year follow-up period, as a result of fewer revision procedures, were observed in patients treated with Coblation.

## Data Availability

This study was based on an economic model that was developed using published clinical aggregate data, the sources of these data are cited in the manuscript.
